# Cholestatic Liver Disease after Rituximab and Adalimumab and the Possible Role of Cross-Reacting Antibodies to Fab 2 Fragments

**DOI:** 10.1371/journal.pone.0078856

**Published:** 2013-11-11

**Authors:** Joerg Latus, Reinhild Klein, Ina Koetter, Matthias Schwab, Peter Fritz, Martin Kimmel, M. Dominik Alscher, Niko Braun

**Affiliations:** 1 Department of Internal Medicine, Division of Nephrology, Robert-Bosch Hospital Stuttgart, Germany; 2 Department of Internal Medicine II, University Hospital Tuebingen, Germany; 3 Dr Margarete Fischer-Bosch-Institute of Clinical Pharmacology and University Tuebingen, Stuttgart, Germany; 4 Institute of Digital Medicine, Stuttgart, Germany; King’s College London, United Kingdom

## Abstract

**Background:**

Millions of patients are treated with therapeutic monoclonal antibodies (Tmabs) for miscellaneous diseases. We investigated sera from six patients who received immune globulin, from one patient with refractory anti-neutrophil-cytoplasmic antibody (ANCA)-associated granulomatosis with polyangiitis (GPA) who developed two episodes of acute cholestatic liver disease, one after treatment with rituximab and a second after adalimumab and a healthy control group.

**Methods:**

Three sera from the patient and six sera from patients who received immune globulin were analyzed for antibodies to rituximab and adalimumab by ELISA. Additionally, sera from the patients and from nine healthy blood donors were coated with the Fab fragment of an unrelated humanized monoclonal antibody, with human Fc proteins as well as a mouse IgG globulin.

**Results:**

Viral serology for hepatitis A, B, C and autoantibodies specific for autoimmune liver disorders were negative. In all three sera from the patient antibodies to rituximab could be detected, but also antibodies to adalimumab were present even at time points when the patient had not yet received adalimumab, indicating cross reactivity between both substances. Testing against an unrelated human Fab fragment revealed positive results, indicating that the patient had antibodies against human Fab fragments in general. The Fc proteins were negative, and patients’ sera did also not react with mouse IgG globulins. Remarkably, 2 out of 5 patients which were treated with immune globulin had antibodies against human Fab fragments in general whereas in none of the samples from healthy controls antibodies to Fab fragment could be detected.

**Conclusion:**

This is the first study demonstrating cholestatic liver disease induced by two different Tmabs. Cross - reacting antibodies to Fab2 fragments in general are probably involved. Further studies must show if these Fab2 antibodies in general are related with drug-induced side effects and accelerated drug clearance in patients on Tmab therapy.

## Introduction

Millions of patients are treated with therapeutic monoclonal antibodies (Tmabs) for miscellaneous diseases. The monoclonal anti CD20 antibody Rituximab is widely used. Rituximab is a chimeric monoclonal antibody against the protein CD20, which is primarily expressed on the surface of B cells. Rituximab is used extensively in leukemias, lymphomas and some autoimmune disorders. Generally, rituximab is well tolerated with toxicity restricted to infusion related reactions causing a syndrome of fever, hypotension, chills and dyspnea [1]. Particularly, there are only two published cases suggesting significant liver toxicity after rituximab infusion [2], [3]. Due to B-cell depletion the risk of infection is increased. There are only a few case reports of fatal infections after treatment with rituximab in the current literature [4]–[7].

Adalimumab, a tumor necrosis factor (TNF) inhibitor is used in patients with e.g. rheumatoid arthritis and several autoimmune disorders. In contrast to rituximab, adalimumab is a fully human monoclonal antibody to TNF. Elevation of liver enzymes is rarely observed after Adalumimab therapy [8].

Both, human antichimeric antibodies (HACA) against rituximab and the formation of human antihuman antibodies (HAHA) are reported in patients with rheumatoid arthritis, systemic lupus erythematodes and Crohn’s disease [9]–[13].

It is becoming increasingly clear, that therapeutic monoclonal antibodies (Tmabs) will elicit an immune response, which may induce adverse effects or reduce efficacy of therapy. In clinical trials, screening for anti-drug immune responses in patients is a regulatory requirement [14] but the measurement of antibodies to HAHA or HACA poses several significant problems due to potential interference with disease related serum factors [15].

Because of treatment failure or side effects, switching therapy to adalimumab, a fully human anti tumor necrosis factor-alpha monoclonal antibody, is often performed. Therefore, adverse effects, interactions or cross-reactivity of these drugs are clinically extremely relevant. It is becoming increasingly clear, that Tmabs will elicit an immune response, which may induce adverse effects or reduce efficacy of therapy.

## Materials and Methods

We included 15 patients in our study. One patient with a remarkably severe course of Granulomatosis and Polyangiitis (GPA) with different second and third line therapies, 5 patients who were previously treated with intravenous immune globulin due to different diseases and 9 healthy blood donors served as controls (baseline characteristics of the study population were shown in table 1). None of the 6 patients in the treatment group received Tmabs prior to immunoglobulin therapy. 3 out of 6 patients received Tmabs after immune globulin treatment. Patients provided prior written informed consent according to an approved protocol (#322/2008BO2, Ethik-Kommission, Eberhard-Karls-Universität Tuebingen, Germany).

**Table 1:** Baseline characteristics of patients treated with intravenous immunoglobulins, SLE systemic lupus erythematodes, PID Primary immunodeficiency disease, ITP Idiopathic thrombocytopenic purpura, MDS myelodysplastic syndrome.

V Variable	Patient 1	Patient 2	Patient 3	Patient 4	Patient 5
**Age**	42	74	90	60	64
**Sex**	Female	Male	Female	Female	Male
**Diagnosis**	SLE	MDS	ITP	ITP	PID
**Intravenous Immuno-** **globulin treatment**	yes	yes	yes	yes	yes
**Treatment with Tmab** **prior to immuno- globulin**	no	no	no	no	no
**Treatment with Tmab** **after immuno- globulin**	no	yes	no	yes	no

**Figure 1:**
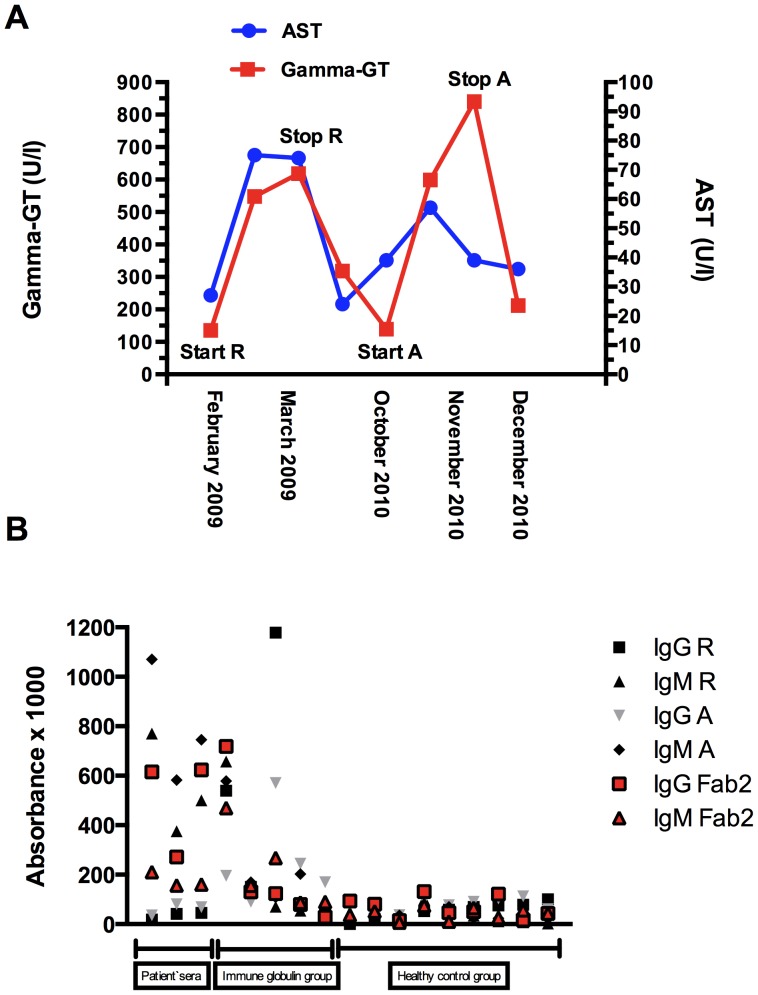
A Time course of serum Gamma-GT and AST levels; R Rituximab, A Adalimumab B Antibody titres of the study population.

This 55 year-old male, has been diagnosed with GPA with pulmonary involvement and positive antibodies to neutrophils (cANCA) and proteinase 3 in March 2007. He was treated initially with i.v. steroid pulse therapy (500 mg for 3 days), followed by 1 mg/kg body weight steroids p.o. and cyclophosphamide i.v. and later p.o. (accumulated dose 35 g). During this therapy the patient developed leukopenia and thrombocytopenia and received immune globulin treatment (30 mg/day for 5 days) in July 2007. Because of refractory disease (Birmingham vasculitis activity score [BVAS] 21) therapy with Rituximab (375 mg/m^2^) was started in March 2008 and repeated in February and March 2009. One month after his fourth dose of Rituximab (March 2009) he was admitted to our nephrology department with weight gain, fatigue, ascites and elevated liver enzymes. Antibodies to neutrophils were slightly elevated (24 RE/ml [normal <20 U/l]). Additionally, he had a thrombocytopenia with 89 GIGA/l [normal 150–370]. There were no signs of any infection and no evidence of hemolytic anemia, no fever. At time of admission BVAS was 18. Rituximab infusions were stopped, liver enzymes normalized spontaneously within 3 weeks. Therapy with adalimumab (40 mg every two weeks subcutaneously) and mycophenolate mofetil (360 mg once a day) was started 7 months later. After adalimumab therapy, the patient was admitted to our hospital again (BVAS 12) due to weight gain, ascites and elevated liver enzymes. Therapy with adalimumab was stopped, liver enzymes normalized spontaneously within 4 weeks and dose of mycophenolate mofetil was increased (360 mg twice a day). The time course of gamma-GT and AST is summarized in figure 1.

### ELISA and Lymphocyte Transformation Test

During the follow up, three sera (from Oct. 2010, Dec 2010, and Sept 2011) from the GPA patient and from 5 patients, which were previously treated with intravenous immune globulin were analysed for antibodies to rituximab and adalimumab by ELISA. For this purpose, Fab2 fragments were prepared from both antibodies using a Pierce Fab preparation kit (Thermo Scientific, Rockford, IL, USA) in order to avoid non-specific binding by the secondary anti-human IgG antibodies. Microtiter plates were coated with these Fab2 fragments at a concentration of 1 µg/ml. Patients’ sera were used at a dilution of 1∶500. As secondary antibodies peroxidase conjugated goat anti-human IgG-Fc antibodies were used (Dianova, Hamburg, Germany; dilution 1∶2000). Results were given as absorbance×1000.

As controls, the microtiter plates were coated with the Fab2 fragments of an unrelated humanized monoclonal antibody, with human Fc proteins (kindly provided by Dr. Gundram Jung and Dr. Ludger Grosse-Harvest, respectively, Department of Immunology, University of Tuebingen) as well as a mouse IgG globulin.

Normal ranges were calculated with sera from nine healthy blood donors. Positive results were defined as mean values of these donors plus twice the standard deviation.

Lymphocyte transformation test using rituximab and adalimumab as antigens was performed applying established methods [16].

## Results

**Figure 2:**
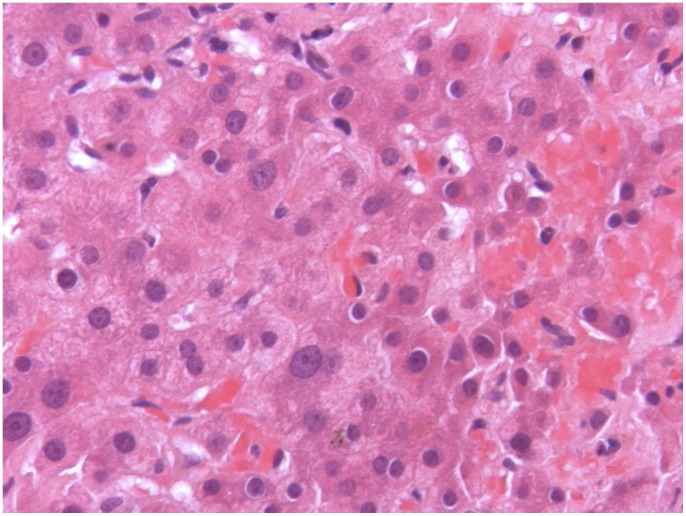
Liver biopsy with scattered foci of cell necrosis and lymphocytic infiltration without signs of involvement of underlying disease.

Extensive work-up was performed in the patient with GPA. Bone marrow biopsy revealed normal marrow and ultrasound of the upper right quadrant showed no mechanical obstructions. Viral serology for hepatitis A, B and C, cytomegalovirus, herpes zoster and Epstein-Barr virus was negative. Autoantibodies specific for autoimmune liver disorders could not be detected, quantitative immunoglobulins were normal. Rheumatoid factor was negative. Transjugular liver bopsy showed drug-induced liver injury (fig. 2). Nine months after the first application of adalimumab a lymphocyte transformation test using rituximab and adalimumab as antigens was performed applying established methods [16] but revealed negative results.

In all three sera (from Oct. 2010, Dec 2010, and Sept 2011) antibodies to rituximab could be detected by ELISA. Also antibodies to adalimumab were present at all three time points, although the patient had not yet received adalimumab in Oct and Dec 2010, indicating possible cross reactivity between both substances. Therefore, patients’ sera were tested against an unrelated human Fab fragment, which also revealed positive results, indicating that the patient has developed antibodies against human Fab fragments in general. The Fc proteins were negative, and patients’ sera did also not react with mouse IgG globulins. Remarkably, 2 out of 5 patients in the immune globulin treatment group showed antibodies against the Fab fragment in general or the Fab fragment of two different Tmabs (Rituximab, Adalimumab). None of the patients in the control group showed antibodies neither to the unrelated human Fab fragment nor to one of the two Tmabs.

## Discussion

There are two major findings of the present study. First, this is the first description of drug-induced hepatic injury after treatment with two different Tmabs. Secondly, we identified antibodies against the Fab2 fragment of both Tmabs, probably responsible for two episodes of acute liver toxicity. A careful clinical work-up revealed GPA activity or infection causing liver deterioration very unlikely but drug induced liver injury more likely. Furthermore, the patient’s rapid recovery after cessation of therapy with rituximab and later adalimumab, and the fact that these substances were the only drugs the patient received in the time preceding the hepatitis episode are strongly suggestive for a drug-induced process. Additionally, liver biopsy showed histological findings compatible with drug-induced injury and no histological findings typical for the underlying disease.

There are only two reports about rituximab-induced liver diseases in the literature. Neither liver biopsy nor antibody testing to rituximab had been performed [2], [3]. The induction of autoimmune hepatitis by adalimumab has been reported in two further cases [17], but again, these patients were not analysed for antibodies to the drug. The mechanism of liver injury in Tmab therapy is not clear. An allergic process can be discussed. However, in our patient no elevation of IgE-globulins or eosinophils was observed. A lymphocyte transformation test with the drugs was performed but revealed negative results. Interestingly, we found antibodies to the Fab2 fragments of rituximab and adalimumab, which were, however, not specific for these substances but rather directed against Fab2 fragments in general. These antibodies were present at three different time points and could not be related to rheumatoid factor (RF), because the patient was RF negative. We used an ELISA to demonstrate these antibodies. This method is often the preferred format to measure antibodies because of its simplicity. Yet, for measuring serum antibodies to Tmabs, the lack of proper secondary reagents that discriminate between serum antibodies and the Tmab form a technical hurdle. One approach to solve this problem, is - as used in the present study – the conversion of the Tmab used in the assay as ‘bait’ for the antibodies, into F(ab’)2 or Fab fragments, allowing the use of secondary reagents reacting with the CH2 and/or CH3 of domain of IgG [15]. Interestingly, these antigenic determinants are only exposed upon coating. However, antibodies reactive with coated F(ab’)2 or Fab fragments have been described also in sera from healthy individuals [18]–[20]. Unfortunately, we had no serum sample from our patient before rituximab therapy so we do not know whether he might have had these antibodies already before treatment or whether they have been really induced by this kind of therapy. Thus, it is important to mention that our patient had received immunoglobulins eight months prior to the rituximab therapy and in sera from two out of five patients with different disorders who had received immune globulin, indeed, we found anti-Fab2 antibodies. One could, therefore, speculate that the anti-Fab2 antibodies in the presented patient had been induced by this therapy. Hence, it will be of interest to analyse the influence of immunoglobulin treatment on the production of anti-Fab2 antibodies in more depth. Furthermore, it is important to investigate whether patients expressing those anti-Fab2 antibodies may be prone to develop adverse reactions towards Tmab treatment.

Development of antibodies to adalimumab has been described in 17% of 121 RA patients in one study [12] and in 6% of 1062 patients in another study [21]. Although there was no cross reactivity with infliximab, a reaction with Fab2 fragments in these instances can not be excluded.

The mechanism by which those antibodies could induce just hepatic injury remains speculative. The formation of circulating immune complexes (IC) has to be considered. Circulating IgG-ICs are normally efficiently eliminated by the reticuloendothelial system of the liver [22]. Whether or not hepatocytes or cholangiocytes are involved in the uptake of IgG-ICs is controversial, although the prevailing opinion is that they are not. Kupffer cells are beyond doubt involved in the process, but also the sinusoidal cells can take part in the uptake of Ig-ICs via Fc receptor interaction [22], [23]. The next unsolved problem implicates the question, whether the antibodies against Fab fragment in general abrogate the pharmacodynamic effect of rituximab and adalimumab. If the drug disappeared much faster than expected relative to his half time, that would argue that these antibodies were resulting in accelerated drug clearance. This might be an explanation for the lower efficacy rates with second and subsequent monoclonal antibody therapy compared to its first use. Concomitant administration of azathioprine, methotrexate or other immunosuppressive agents might prevent from this effect.

In conclusion, this is the first presentation of a patient developing hepatic injury after two different Tmab. In this respect, the presence of antibodies to Fab2 fragments of rituximab and adalimumab cross reacting also with unrelated Fab2 fragments in the patient’s sera is of interest but whether they are responsible for the adverse reaction cannot be concluded with certainty. Therefore, further studies are mandatory to investigate whether Fab2 antibodies are related with drug-induced side effects or whether these antibodies result in accelerated drug clearance causing lower efficacy rates with second and subsequent monoclonal antibody therapy.
